# Co-activation of Taxonomic and Thematic Relations in Spoken Word Comprehension: Evidence From Eye Movements

**DOI:** 10.3389/fpsyg.2019.00964

**Published:** 2019-05-03

**Authors:** Pingping Xu, Qingqing Qu, Wei Shen, Xingshan Li

**Affiliations:** ^1^Key Laboratory of Behavioral Science, Institute of Psychology, Chinese Academy of Sciences, Beijing, China; ^2^Department of Psychology, University of Chinese Academy of Sciences, Beijing, China; ^3^Institute of Psychological Sciences, Hangzhou Normal University, Hangzhou, China; ^4^Zhejiang Key Laboratory for Research in Assessment of Cognitive Impairments, Hangzhou, China; ^5^Centre for Cognition and Brain Disorders, Hangzhou Normal University, Hangzhou, China

**Keywords:** semantic system, taxonomic relation, thematic relation, co-activation, visual-world paradigm

## Abstract

Evidence from behavior, computational linguistics, and neuroscience studies supported that semantic knowledge is represented in (at least) two semantic systems (i.e., taxonomic and thematic systems). It remains unclear whether, and to what extent taxonomic and thematic relations are co-activated. The present study investigated the co-activation of the two types of semantic representations when both types of semantic relations are simultaneously presented. In a visual-world task, participants listened to a spoken target word and looked at a visual display consisted of a taxonomic competitor, a thematic competitor and two distractors. Growth curve analyses revealed that both taxonomic and thematic competitors attracted visual attention during the processing of the target word but taxonomic competitor received more looks than thematic competitor. Moreover, although fixations on taxonomic competitor rose faster than thematic competitor, these two types of competitors started to attract more fixations than distractor in a similar time window. These findings indicate that taxonomic and thematic relations are co-activated by the spoken word, the activation of taxonomic relation is stronger and rise faster than thematic relation.

## Introduction

The structure and organization of semantic knowledge are critical to nearly all aspects of human cognition such as object recognition, memory and language processing. Evidence from behavior, computational linguistics, and neuroscience studies supported that semantic knowledge is represented in (at least) two semantic systems (see Mirman et al., 2017 for a review): a taxonomic system in which semantic knowledge is organized based on categories that are defined by shared semantic features such as fruit (e.g., Collins and Loftus, 1975), and a thematic system with the organization of semantic information based on events or scenarios such as objects involved in building a house (e.g., Estes et al., 2011). For instance, mouse and dogs are taxonomically related because they share semantic features; mouse and cheese are thematically related because they often co-occur in the same scenario or event, but they do not have common features.

Behavioral studies demonstrating a dissociation between taxonomic and thematic semantics widely used semantic judgement or related semantic tasks. In a “triads” task, participants are asked to choose which of two options is most related to a target. This task has revealed qualitatively different patterns of effects for various concept domain: taxonomic relation tends to be more important for natural objects such as animals, whereas thematic relation is more important for manipulable objects such as tools (Bonthoux and Kalénine, 2007; Kalénine et al., 2009). More evidence came from the domain of spoken word production. In a picture-word interference task in which semantic relations between picture names and distractor words were manipulated, de Zubicaray et al. (2013) demonstrated that taxonomic similarity between picture names and distractor words inhibited picture naming whereas thematic relation facilitated picture naming. These findings indicate that taxonomic and thematic semantic relations are functionally distinct.

A number of studies have demonstrated the neural basis dissociation between taxonomic and thematic relations. In a large-scale study of spoken errors in picture naming produced by adults with aphasia, Schwartz et al. (2011) showed that various forms of brain lesion independently influenced semantic errors: Aphasias with lesions in the left anterior temporal lobe (ATL) appeared to produce a higher proportion of taxonomic errors (“pear” in response to apple), and those with lesions in the left temporoparietal junction (TPJ) caused more thematic errors (“worm” in response to apple). Based on these results, Schwartz et al. argued that taxonomic relation with ATL as the critical hub, and thematic relation with TPJ as the critical hub are complementary semantic systems. Although there is less consensus on the precise neural basis underlying taxonomic and thematic semantics, there is general agreement about the neural dissociation of taxonomic and thematic semantics (see Mirman et al., 2017 for a review).

More recently, eye tracking technique has opened up a new avenue for investigating semantic knowledge. In a visual world paradigm, participants listen to a spoken utterance while viewing a visual display, and their eyes are tracked (Cooper, 1974; Tanenhaus et al., 1995). Adopting this task, Mirman and Graziano (2012) demonstrated that both types of semantic knowledge were activated, with larger effect for taxonomic relation. Interestingly, individuals' relative strength of taxonomic relation vs. thematic relation in eye-tracking measurement could predict individuals' preference on taxonomic relation over thematic relation in triads task, which has been taken to indicate that individuals differ in the relative strengths of both types of semantic knowledge. In sum, taxonomic and thematic relations differentially contribute to semantic systems as demonstrated in studies above.

Moreover, there is evidence that taxonomic and thematic relations differ in the time courses of activation. Time courses of these two semantic effects have been assessed in eye-tracking visual world task which does not involve metalinguistic decision, and thus has strengths than widely used semantic priming or judgement tasks. Kalénine et al. (2012) observed that thematic relation produces earlier and more transient effects than artificial taxonomic relation. A subsequent EEG study (Wamain et al., 2015) combining the semantic priming task demonstrated that early ERP components (N1 and P3) were only sensitive to thematic relation, and late N400 component was modulated by both semantic relations, reflecting earlier activation of thematic representations compared to taxonomic representations. However, results from recent behavioral research have demonstrated that activation of taxonomic and thematic relations may proceed in parallel. For example, Jones and Golonka (2012) compared taxonomic and thematic relations (along with integrative relation, see Estes and Jones, 2009; Jones et al., 2017 for integrative relation) in a lexical decision task across three SOAs (100, 500 or 800 ms), in which participants judged whether the target (following integrative, thematic taxonomic or unrelated prime) was a real word or not. Results showed no difference in strength or time course of priming between thematic and taxonomic relations.

In the visual-world studies reviewed above, the two types of semantic competitors were presented on separate displays, as in the classical version of the visual world paradigm and other relevant priming tasks, which caused an indirect way to assess the strength and timing of these two types of relations. It remains unclear whether and to what extent taxonomic and thematic relations are co-activated when both types of semantic relations are simultaneously presented on a trial and measured by the online method (VWP). In the present study, we presented the two critical types of semantic competitors within a single visual display, with the aim to investigate whether the two types of semantic relations are co-activated. If this is the case, what is the relative strength and timing of taxonomic vs. thematic relations? Moreover, in previous studies, target objects were often co-present in the visual scene. When target objects are co-present in the visual display, most of fixations tend to shift toward these target objects, causing less fixations toward the other objects (Huettig and Altmann, 2005). In order to maximally capture looking behavior driven by semantic relations, we presented the targets verbally only and not visually to explore the activation of semantic relations when targets are not presented in visual display. In the experiment, participants listened to a spoken target word while viewing a visual display of four objects consisting of two competitors and two distractors. Fixation proportions on different types of objects in continuous time courses reflect participants' activation of the semantic relations. Based on the studies reviewed above, we expected both of the taxonomic and thematic competitors to attract more fixations than unrelated distractors. The more interesting question was whether the relative strength and time courses of activation of taxonomic vs. thematic relations differ.

## Methods

### Participants

Twenty-five native Chinese speakers (9 females, age 20–30 years, mean age 23 years) participated in the experiment. They were undergraduate students from universities in Beijing, China, and were paid RMB 20 (about 3.5 US $) for their participation. All participants had normal or corrected-to-normal vision. This study was approved by the ethics committee of the Institute of Psychology, Chinese Academy of Sciences. We obtained written informed consent from the participants of this study.

### Materials and Design

**Figure 1:**
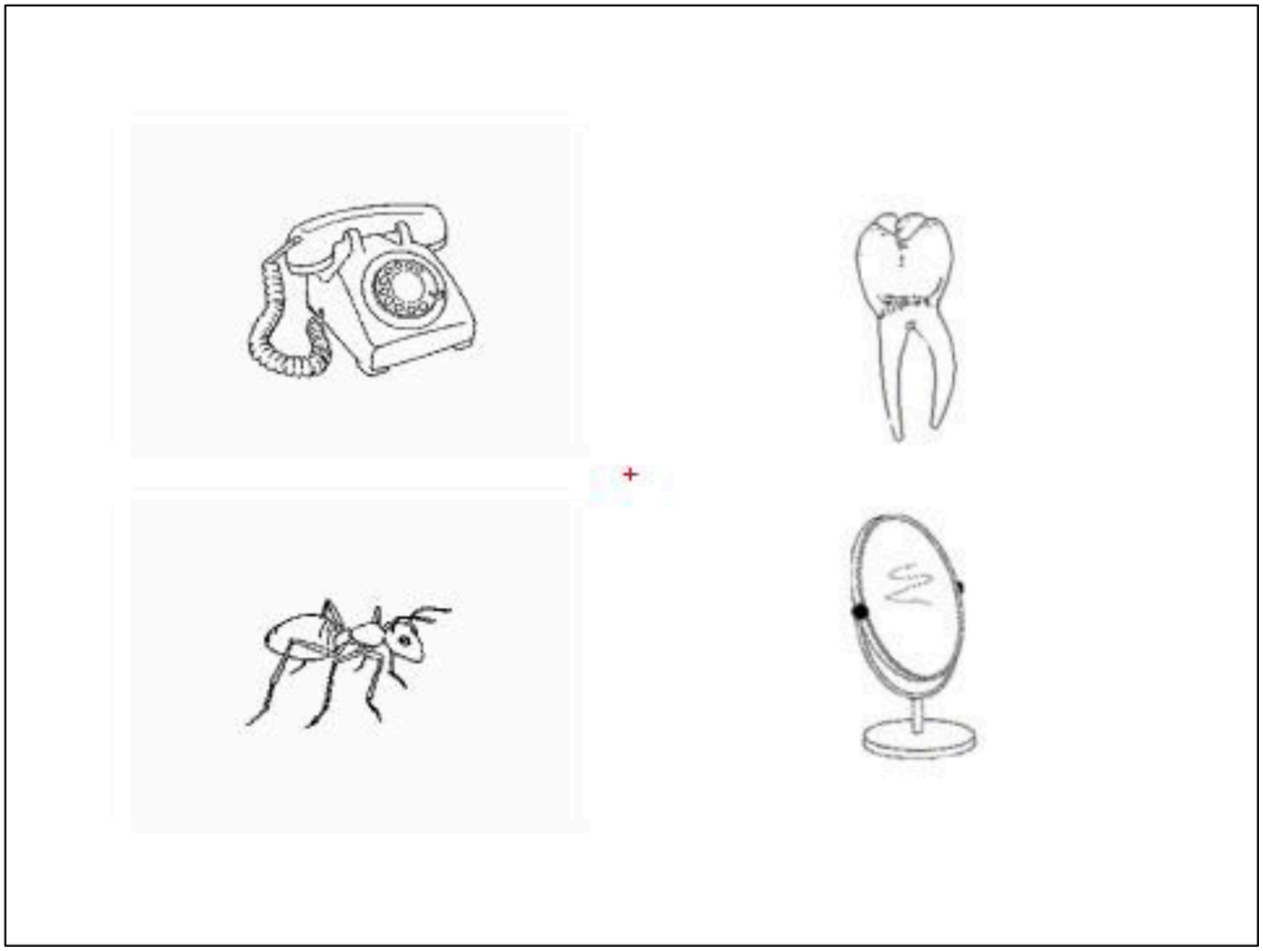
For the spoken word “耳朵” (er3duo, “ear”), the visual display consisted of a taxonomic competitor “牙齿” (ya2chi3, “tooth”), a thematic competitor object “电话” (dian4hua4, “phone”) and two unrelated distractors, “蚂蚁” (ma3yi3, “ant”), and “镜子” (jing4zi, “mirror”).

Forty words were selected as the spoken target words to construct 40 visual displays, but four of these sets were eliminated for the reason of counterbalance of object positions, leaving 36 sets of critical items. All spoken words were recorded in a natural tone by a female native Chinese speaker. The mean duration of spoken words was 689 ms (ranged from 539 to 836 ms, *SD* = 76). Each visual display consisted of four pictures of common objects, one taxonomic competitor, one thematic competitor and two unrelated distractors (see Figure 1 for an example). The competitors were selected under criteria that the taxonomic competitor was as low as possible in thematic relatedness with the target and the thematic competitor was as low as possible in taxonomic relatedness with the target. Moreover, all word pairs were phonologically or orthographically unrelated (see Appendix 1 for a complete list of stimuli). The positions of the four pictures were fully counterbalanced in the display. In addition, across experimental conditions, the names of objects in displays were matched closely on word frequency, name agreement, and naming response time (*F*s < 1, *p*s > 0.40; see Table 1 for the properties of the experimental materials).

**Table 1:** Properties and mean rating scores across conditions (standard deviations in parentheses).

	**Thematic competitor**	**Taxonomic competitor**	**Distractor1**	**Distractor2**
Mean word frequency (occurrence per million)	12.12 (30.39)	8.56 (12.70)	6.75 (14.42)	7.82 (17.65)
Name agreement (%)	67.53 (21.17)	72.78 (22.79)	64.44 (18.72)	68.50 (21.99)
Naming response time (ms)	1,019 (231)	1,070 (210)	1,064 (205)	1,039 (222)

Three subjective rating studies were conducted to evaluate the general semantic relatedness, taxonomic relatedness and thematic relatedness between the spoken target and the four objects within one set. The first rating asked participants to judge the general semantic relatedness between word pairs (either taxonomically or thematically related). The second rating asked participants to judge the taxonomic relatedness between word pairs, i.e., the degree of being in the same taxonomy. The third rating asked participants to judge the taxonomic relatedness between word pairs, i.e., the degree of connected via events or scenarios. All of the rating were based on a 7-point scale (1 = not related at all, 7 = strongly related), each collected rating scores form different sets of 17 participants. We followed the instructions (Appendix 2) used in Jones and Golonka (2012). Each rating contained all of the spoken targets, paired with the two competitors and two distractors, which were presented randomly. The means, SDs, minimums, and maximums on each of the three rating tasks are shown for each rating in Table 2. Separate One-way ANOVAs and LSD *post-hoc* tests (see Table 3) on the rating scores confirmed that: (1) both types of competitors were rated higher semantically related with the spoken target than the distractors, rating scores did not differ significantly between the two competitors, neither between the two distractors, (2) taxonomic competitor was highest rated in taxonomic relatedness rating, follow by thematic competitor, and two distractors were rated comparably low, and (3) thematic competitor was highest rated in thematic relatedness rating, follow by taxonomic competitor, and two distractors were rated comparably low. These rating results suggest that the selection of material was valid.

**Table 2:** Means, Standard Deviations, Minimums, and Maximums of ratings.

**Rating**	**Taxonomic competitor**				**Thematic competitor**				**Distractor 1**				**Distractor 2**			
	**M**	**SD**	**Min**	**Max**	**M**	**SD**	**Min**	**Max**	**M**	**SD**	**Min**	**Max**	**M**	**SD**	**Min**	**Max**
General relatedness	6.27	0.41	5.28	6.92	5.86	0.79	3.14	6.72	1.34	0.38	1.00	2.56	1.41	0.41	1.00	2.61
Taxonomic relatedness	6.11	0.60	4.61	6.75	4.09	1.16	2.22	5.75	1.35	0.34	1.00	2.06	1.38	0.36	1.00	2.47
Thematic relatedness	3.52	1.32	1.33	6.56	5.20	0.66	4.11	6.56	1.19	0.17	1.00	1.58	1.23	0.20	1.00	1.67

**Table 3:** Differences among objects for each rating.

**Rating**	**ANOVA**	**Comparison (*Post-hoc*, LSD)**
General relatedness	*F* = 456.22, *p* < 0.001	Taxonomic = Thematic >Distractor 1 = Distractor 2
Taxonomic relatedness	*F* = 185.96, *p* < 0.001	Taxonomic >Thematic >Distractor 1 = Distractor 2
Thematic relatedness	*F* = 114.51, *p* < 0.001	Thematic >Taxonomic >Distractor 1 = Distractor 2

Besides, 40 filler trials were added. Each filler set consisted of four objects with no overlap in meaning and visual form. One of the four objects was randomly selected as the target word. In total, 304 pictures were used, 218 of which were selected from the data base developed by Liu et al. (2011), and the other 86 were from Severens et al. (2005). All of the pictures were black-and-white line drawings and adjusted to the same size of 350 × 250 pixels and of approximately 13.67° × 9.77° visual angle.

### Apparatus

Eye movements were recorded using an EyeLink 1000 tracker (SR Research, Mississauga, Ontario, Canada). Experimental materials were presented on a 21-in. CRT monitor (Sony Multiscan G520) with a 1,024 × 768 pixels resolution and a refresh rate of 150 Hz. The eye-tracking system was sampled at 1000 Hz. The participants placed their chins on a chin-rest and leaned their foreheads on a forehead rest to minimize head movements. Although viewing was binocular, eye movement data were collected only from the right eye. Participants were seated 58 cm from the video monitor.

### Procedure

Participants were first asked to familiarize themselves with the pictures by viewing them in a booklet, with the name printed underneath each picture. Subsequently, the eye tracker was calibrated and validated, and re-calibration was conducted whenever the error was >1° during the experiment. The participants performed this procedure by means of a nine-point calibration, and the validation error was smaller than 0.5° of visual angle on average. A drift check was performed at the beginning of each trial, and then a blank screen was presented for 600 ms. The visual display was presented 2,000 ms before the onset of the spoken target word. Spoken target words were presented to participants through a headphone (Philips, SHM6110, China). Participants were asked to listen to the spoken words and view the displayed pictures without performing any explicit task. The visual display disappeared 4,000 ms after the spoken word onset. Each participant performed 36 critical and 40 filler trials which were randomly intermixed, following 8 practice trials. Trials were presented in a random order, and the entire experiment lasted approximately 20 min.

## Results

**Figure 2:**
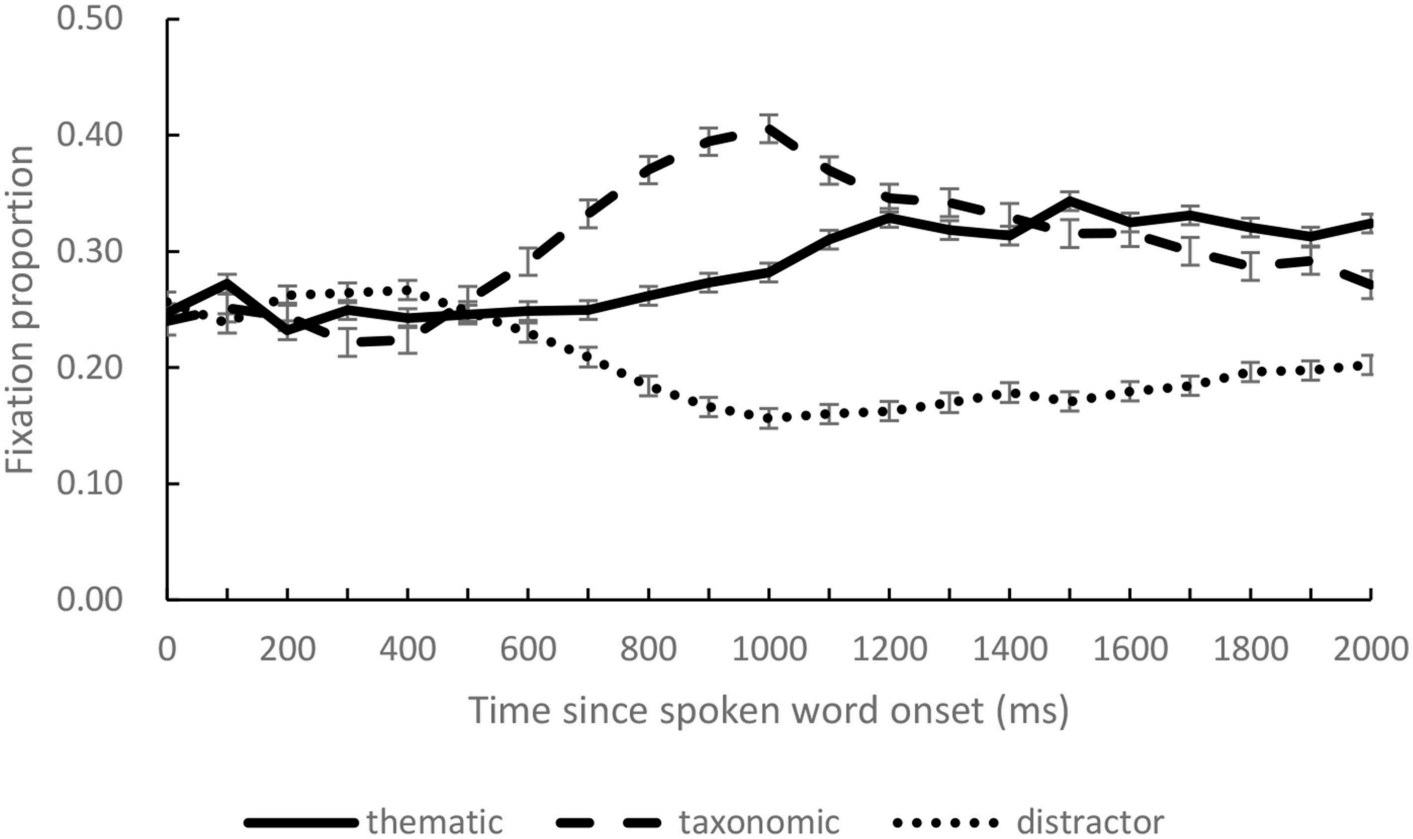
The fixation proportions on thematic competitor, taxonomic competitor, and distractor objects in the critical time window (0 on the x-axis indicates the onset of the spoken target words).

Four areas of interest were defined, each overlapped with one of the pictures, corresponding to a rectangle (350 × 250 pixels). We calculated the mean fixation proportions of taxonomic competitor, thematic competitor, and distractors over successive 100 ms intervals. Figure 2 presents the distributions of the mean fixation proportions to each condition (distractor averaged from fixations on two distractors) from the onset of the target to 2,000 ms after the onset of the spoken target word.

We adopted the growth curve analysis (GCA), a multilevel regression modeling technique using fourth-order orthogonal polynomials (Mirman et al., 2008), to quantify the effects of semantic relations. Four terms are included in such analyses, the intercept term represents the mean proportion of fixations over the entire window, the linear term captures variation in how rapidly looks to an object rise over time, the quadratic term captures variation in the curvature of the line representing looks to each object, and the cubic and quartic terms reflect the inflections at the extremities of the curve (Kalénine et al., 2012; Gambi et al., 2016). All analyses were carried out in R version 3.3.2 using the lme4 package (version 1.1-17). The first model compared fixed effects of object type (taxonomic competitor, thematic competitor and unrelated distractor) on all time terms, with unrelated distractors as the baseline, which compared the two types of competitors with the distractors respectively. The model also included random effects of participants on all time terms, which were captured with the crossed random effects structure, which estimates the random effects of participant and participant: object type (Mirman, 2014, p 70)^1^. We analyzed fixation proportions during the time window of 500–1,000 ms, which started from the earliest onset (539 ms) and ended at the latest offset of spoke target word (836 ms) plus about 200 ms (needed to programme and launch an eye movement, e.g., Hallett, 1986). This time window reflected the online processing of the spoken word. When a model includes three levels of a variable, the model first assessed baseline (Mirman, 2014, p. 95) and showed significant intercept (estimate = 0.198, *p* < 0.001) and linear (estimate = −0.078, *p* < 0.001) terms. Importantly, paired comparisons showed that both types of semantic competitors received more fixations than distractors (for intercept term, taxonomic vs. distractor: estimate = 0.144, *p* < 0.001; thematic vs. distractor: estimate = 0.062, *p* < 0.001). Effects on linear term were also significant, suggesting that fixation rose faster on both of the competitors than distractors (taxonomic vs. distractor: estimate = 0.207, *p* < 0.001; thematic vs. distractor: estimate = 0.109, *p* < 0.001) (see Table 4 for details).

**Table 4:** Parameter Estimated for Analysis of Effect of Object Type (first model).

	**Estimate**	***SE***	***t***	***p***
Intercept	0.198	0.007	29.26	<0.001
Linear	−0.078	0.010	−7.99	<0.001
Quadratic	0.006	0.007	0.92	0.36
Cubic	0.006	0.006	1.01	0.31
Quartic	0.0005	0.006	0.08	0.94
Taxonomic: intercept	0.144	0.017	8.51	<0.001
Taxonomic: linear	0.207	0.026	8.08	<0.001
Taxonomic: quadratic	−0.026	0.013	−1.94	0.06
Taxonomic: cubic	−0.016	0.015	−1.11	0.27
Taxonomic: quartic	0.002	0.012	0.15	0.88
Thematic: intercept	0.062	0.014	4.35	<0.001
Thematic: linear	0.109	0.019	5.62	<0.001
Thematic: quadratic	0.001	0.015	0.10	0.92
Thematic: cubic	−0.009	0.010	−0.90	0.37
Thematic: quartic	−0.003	0.011	−0.31	0.75

In the second model, we compared the fixation proportions on taxonomic and thematic competitors, with taxonomic competitors as the baseline. This model revealed a significant difference (intercept term, estimate = 0.342, *p* < 0.001; linear term, estimate = 0.130, *p* < 0.001). Paired comparison between the two competitors showed significant intercept term, estimate = −0.082, *p* < 0.001, indicating more fixations on the taxonomic than the thematic competitor. The linear term suggested that fixations on taxonomic competitor rose faster than that on the thematic competitor (estimate = −0.098, *p* < 0.001). Moreover, other inflections on the time courses of taxonomic and thematic effects did not differ significantly, as suggested by the quadratic, cubic and quartic terms (see Table 5 for details).

**Table 5:** Parameter Estimated for Analysis of Effect of Object Type (second model).

	**Estimate**	***SE***	***t***	***p***
Intercept	0.342	0.012	29.65	<0.001
Linear	0.130	0.018	7.04	<0.001
Quadratic	−0.019	0.009	−2.07	0.07
Cubic	−0.010	0.011	−0.91	0.36
Quartic	0.002	0.008	0.27	0.78
Thematic: intercept	−0.082	0.016	−4.99	<0.001
Thematic: linear	−0.098	0.027	−3.62	<0.001
Thematic: quadratic	0.027	0.015	1.79	0.07
Thematic: cubic	0.007	0.016	0.45	0.66
Thematic: quartic	−0.005	0.012	−0.43	0.67

**Figure 3:**
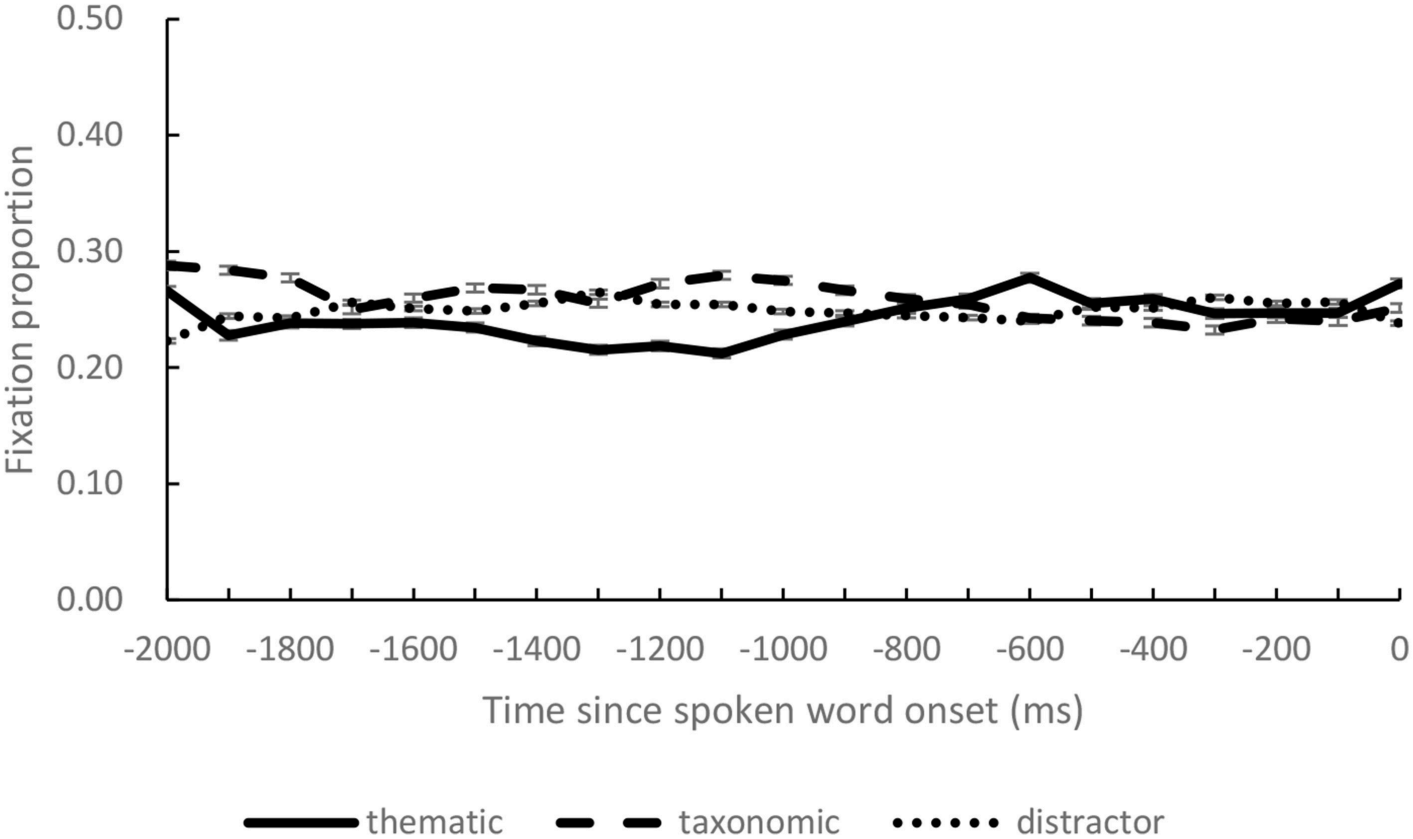
The fixation proportions on thematic competitor, taxonomic competitor, and distractor objects during preview (0 on the x-axis indicates the onset of the spoken target words).

Besides the critical time window, we also calculated fixation proportions over successive 100 ms intervals during the preview period to test if there was any preference on the pictures caused by factors other than the spoken activation. We compared fixation proportions on the four objects in the same way as the first model in analysis of critical time window. The results suggested that fixation proportions on both of the competitors did not differ significantly from that on the distractors (taxonomic competitor: estimate = 0.011, *p* = 0.35; thematic competitor: estimate = −0.008, *p* = 0.36). Actually, during the preview period, fixation proportions on the four objects approximated the chance level 0.25 (see Figure 3).

## Discussion

The present study examined the semantic representations invoked by a spoken word when the referent object was absent in the visual scene. Two types of semantic relations were compared, which are proposed as complementary systems and investigated widely (Mirman et al., 2017). We found that both the taxonomic and thematic competitors were fixated more than the unrelated distractors, indicating that the spoken input induced activation of these semantic representations. Rise of fixation proportion on taxonomic and thematic competitors started at similar time points, about 500 ms after the spoken word onset.

The finding that the magnitude of taxonomic effect and the rise speed of fixations on the taxonomic competitor was greater than the thematic effect was consistent with findings with the previous studies in which target objects were presented in visual displays (e.g., Mirman and Graziano, 2012). Mirman and Graziano (2012) proposed three possibilities for the stronger taxonomic effects relative to thematic effects: first, in their studies, the taxonomic competitors were also thematically related eliciting stronger semantic relation in taxonomic pairs; Second, since taxonomic relation is defined by shared semantic features, taxonomic competitors highly possibly shared visual features with the targets, which would cause more visual attention, as suggested by the finding that competition effects for shape similarity are earlier than for non-perceptual similarity (Yee et al., 2011); and Third, recognition of concrete objects used in their experiments was dominated by taxonomic knowledge (see also Duñabeitia et al., 2009), which resulted in stronger activation of taxonomic relation. In the present study, ratings on semantic relatedness suggested that taxonomic competitor and thematic competitor did not differ in general semantic relatedness, which rules out the possibility that taxonomic competitors are stronger in semantic relatedness. We speculate that visual similarity between taxonomic competitor and the target could have caused the larger taxonomic effect in the present study. Visual similarity naturally arises in taxonomically related items. As Mirman and Graziano (2012) emphasized, it is not practical to choose taxonomic competitor not visually similar with the target, Moreover, all of the concepts we used are concrete, and such concreteness of the stimuli used in the present study could also be responsible for the larger magnitude. This explanation is in agreement with the argument that concrete words are organized based on semantic features rather than association (Duñabeitia et al., 2009).

What could be the reason for the similar start points of two effects, in contrast to an earlier thematic effect relative to a taxonomic effect previously found by Kalénine et al. (2012)? Differences in experimental stimuli could be one potential reason. The stimuli used in the current study were mixed with natural and man-made objects, whereas all stimuli in Kalénine et al. were man-made objects. It is well-established that the role of taxonomic relation and thematic relation differ by concept domain, with thematic relation more important for artifacts/man-made/manipulable objects and taxonomic relation more important for natural objects (Bonthoux and Kalénine, 2007; Kalénine et al., 2009). The stimuli of artifacts such as tools used in Kalénine et al. could elicit earlier thematic effects, whereas the combined usage of natural and man-made objects could elicit the synchronization of taxonomic and thematic effects in the present study.

One of novelty in the current study was to adopt target-absent visual displays (Huettig et al., 2011 for a review). We provide evidence for activation of both types of semantic relations when the target was absent, as is the case in studies with target objects presented in the visual display (e.g., Kalénine et al., 2012; Mirman and Graziano, 2012), hence arguing for the idea that the semantic activation of a word does not depend on the its visual referent, but on the representations in mental lexicon. The activation invoked by spoken input without visual referent was also observed in children. Swingley and Fernald (2002) found that 2-year-old children maintained their fixation if the fixated object was the exact referent of what they heard, but shifted their gaze and searched other objects if the spoken word could not apply to the fixated object. These findings were interpreted as evidence that by 24 months, rapid activation in word recognition does not depend on the presence of the words' referents. Rather, very young children are capable of quickly and efficiently interpreting words in the absence of visual supporting context. Visual attention shift occurred because the visible objects did not match the representation activated by the spoken word. A recent auditory priming study (Willits et al., 2013) found semantic priming effect with bare spoken word stimuli (e.g., shorter looking time for “kitty-kitty-kitty” followed semantic prime “dog-dog-dog” than unrelated word “juice-juice-juice”), suggesting that young children activate lexical semantic knowledge in the absence of visual referents or sentence contexts. The present study, with adult participants, further suggests that spoken word invoked multiple semantic representations, reflected by more attention paid on the taxonomic and thematic related objects, when the visual display did not contain the target object.

In the present study, taxonomic and thematic competitors were depicted within the same visual display and both attracted more fixations than the unrelated distractors. As we mentioned in the Introduction section, taxonomic and thematic relations are found to be dissociated both in behavioral and neural studies (e.g., Bonthoux and Kalénine, 2007; Kalénine et al., 2009; de Zubicaray et al., 2013). In the perspective of language function, taxonomic relation contributes to organize classes of objects, concepts, and even people appropriately based on features, while thematic relation conveys knowledge about events and scenarios, which help us to establish expectation about upcoming events and complement one's knowledge about features and taxonomic relations (Estes et al., 2011). The present finding implies that although located in different hubs, taxonomic and thematic relations are activated by the same linguistic input, which might help us to establish a more complete representation of the intended concept.

In conclusion, the present study suggested that taxonomic and thematic relations are co-activated by the spoken word, even when the referent object is absent from the visual context. These two relations are activated at similar time points, but the taxonomic effect is stronger and develops faster than the thematic relation in the processing of concrete concepts.

## Ethics Statement

All procedures performed in studies involving human participants were in accordance with the ethical standards of the institutional and/or national research committee and with APA regulations.

## Author Contributions

PX, QQ, and XL inspired the idea of the study, designed the experiment, collected the data, conducted the data analysis, interpreted the results, and wrote the manuscript. WS provided the instruction to the execution of the experiment and data analysis. All authors agreed on the final version of the manuscript.

### Conflict of Interest Statement

The authors declare that the research was conducted in the absence of any commercial or financial relationships that could be construed as a potential conflict of interest.

## Footnotes

^1^R syntax for the models (model 1 and 2 used the same syntax, differed in the content of data): t <-poly(unique(fix$timeBin),4) fix[,paste(“ot”,1:4,sep = "“)] <- t[fix$timeBin,1:4] m.full1 <-lmer(meanFix~(ot1+ot2+ot3+ot4)^*^Object + ((ot1+ot2+ot3+ot4)^*^Object | Subject), control = lmerControl(optimizer = ”bobyqa"), data = fix, REML = FALSE)”

## Appendix 1: Stimuli Used in the Critical Trials

**Table:**

**Target**	**thematic**	**taxonomic**	**distractor1**	**distractor2**
靶子 target	箭头 arrow	飞镖 dart	水母 jellyfish	草莓 strawberry
衬衫 shirt	熨斗 iron	西装 suit	算盘 abacus	飞机 airplane
耳朵 ear	电话 phone	牙齿 tooth	蚂蚁 ant	镜子 mirror
法官 judge	假发 wig	警察 police	土豆 potato	排球 volleyball
肥皂 soap	毛巾 towel	唇膏 lipstick	天线 antenna	手臂 arm
钢琴 piano	凳子 stool	圆号 French horn	书包 backpack	气球 balloon
公园 garden	长凳 bench	教堂 church	水桶 bucket	篮子 basket
火箭 rocket	月亮 moon	大炮 cannon	浴缸 bathtub	蜜蜂 bee
火腿 ham	屠夫 butcher	烤肉 roast	书架 shelf	靴子 boots
橘子 orange	果树 fruit tree	菠萝 pineapple	天平 scale	扑克 poker
锯子 saw	木头 wood	电钻 drill	篮球 basketball	蝴蝶 butterfly
烤炉 oven	厨师 cook	冰箱 refrigerator	螳螂 mantis	地图 map
裤子 pants	皮带 belt	毛衣 sweater	海螺 conch	大脑 brain
老鼠 rat	奶酪 cheese	刺猬 hedgehog	椅子 chair	小丑 clown
领带 tie	别针 pin	项链 necklace	蛋糕 cake	小鸡 chicken
笼子 cage	小鸟 bird	铁链 chain	玫瑰 rose	地毯 rug
萝卜 carrot	兔子 rabbit	南瓜 pumpkin	水井 well	扳手 wrench
骆驼 camel	沙漠 desert	山羊 goat	弹弓 catapult	芹菜 celery
绵羊 sheep	农场 farm	奶牛 cow	樱桃 cherry	电脑 computer
蘑菇 mushroom	白云 cloud	玉米 corn	仙鹤 crane	拐杖 crutch
闹钟 alarm clock	铃铛 bell	日历 calendar	钻石 diamond	螃蟹 crab
纽扣 button	外套 coat	拉链 zipper	杠铃 barbells	蚯蚓 earthworm
女巫 witch	扫帚 broom	神父 priest	光盘 CD	蜻蜓 dragonfly
裙子 skirt	女孩 girl	背心 vest	鸡蛋 egg	大象 elephant
生菜 lettuce	树叶 leaf	丝瓜 loofah	眼睛 eye	鱼缸 fish tube
书桌 desk	铅笔 pencil	沙发 sofa	柠檬 lemon	豹子 leopard
梳子 comb	头发 hair	牙刷 toothbrush	窗户 window	酒杯 wineglass
司机 driver	轿车 saloon car	医生 doctor	哨子 whistle	野猪 pig
铁轨 rail	火车 train	公路 highway	松鼠 squirrel	手套 glove
围巾 scarf	脖子 neck	帽子 hat	地球 globe	金鱼 goldfish
卧室 bedroom	窗帘 curtain	厨房 kitchen	母鸡 hen	头盔 helmet
蜥蜴 lizard	石头 rock	恐龙 dinosaur	水壶 kettle	绳子 rope
香蕉 banana	猴子 monkey	葡萄 grape	马桶 toilet	雨伞 umbrella
小号 trumpet	乐队 band	笛子 flute	手表 watch	喷壶 watering can
包裹 package	礼物 present	盒子 box	企鹅 penguin	花生 peanut
油漆 paint	刷子 brush	颜料 pigment	枕头 pillow	图钉 pushpin

## Appendix 2: Instructions for Rating Studies

### General Relatedness

请判断下列词对之间的语义相关性, 1表示无关, 7表示非常相关。请用1-7（（数字1, 2, 3, 4, 5, 6, 7））进行评定。语义相关性包括类别相关（属于同一个类别的词对, 如蝴蝶-蜜蜂都是昆虫, 应该评为高相关, 蝴蝶-筷子分别属于不同类别, 应该评为低相关）和主题相关性（两个词虽然不是同一类事物, 但是可以通过事件、功能或场景产生联系, 如螃蟹-大海, 伤口-绷带都应该评定为高相关, 螃蟹-考试, 伤口-水杯为低相关）。

Please rate the semantic relatedness between each of the following word pairs on a scale from 1 (not thematically connected) to 7 (highly thematically connected). Please use the full range of the scale (1, 2, 3, 4, 5, 6, or 7) in indicating your responses. Semantic relatedness includes taxomomical relatedness (two words belong to the same specific category, e.g., BUTTERFLY and BEE are both types of insect, thus are taxonomically connected, whereas BUTTERFLY and CHOPSTIC belong to different categories) and thematic relatedness (the concepts are linked together in a common scenario, event, or function, e.g., CRAB-SEA and WOUND-BANDAGE are both highly related, whereas CRAB-EXAM and WOUND-CUP are lowly related).

### Taxonomic Relatedness

请判断下列词对之间的类别相关性, 1表示无关, 7表示非常相关。请用1-7（（数字1, 2, 3, 4, 5, 6, 7））进行评定。类别相关性是指两个词语所代表的事物属于同一类别的程度, 如蝴蝶-蜜蜂都是昆虫, 应该评为高相关。蝴蝶-筷子分别属于不同类别, 应该评为低相关。

Please rate the taxonomic relatedness between each of the following word pairs on a scale from 1 (not thematically connected) to 7 (highly thematically connected). Please use the full range of the scale (1, 2, 3, 4, 5, 6, or 7) in indicating your responses. Taxonomic relatedness refers to the extent to which the two words belong to the same specific category. For example, BUTTERFLY and BEE are both types of insect, thus these items are taxonomically connected. Whereas BUTTERFLY and CHOPSTIC belong to different categories.

### Thematic Relatedness

请判断下列词对之间的类别相关性, 1表示无关, 7表示非常相关。请用1-7（（数字1, 2, 3, 4, 5, 6, 7））进行评定。类别相关性是指两个词语所代表的事物属于同一类别的程度, 如蝴蝶-蜜蜂都是昆虫, 应该评为高相关。蝴蝶-筷子分别属于不同类别, 应该评为低相关。

Please rate the thematic relatedness between each of the following word pairs on a scale from 1 (not thematically connected) to 7 (highly thematically connected). Please use the full range of the scale (1, 2, 3, 4, 5, 6, or 7) in indicating your responses. Thematic relatedness refers to the extent to which the concepts are linked together in a common scenario, event, or function. For example, CREAM is often added to COFFEE, and CHALK is used to write on a BLACKBOARD. Thus, these items are thematically connected. Please note that thematically connected items may not necessarily share similar features. For example, BLACKBOARD and CHALK are different shapes and sizes and are made out of different materials.
